# Tamsulosin vs. Tadalafil as medical expulsive therapy for distal ureteral stones: a systematic review and meta-analysis

**DOI:** 10.1590/S1677-5538.IBJU.2023.0345

**Published:** 2024-02-07

**Authors:** Mikhael Belkovsky, Giulia Veneziani Zogaib, Carlo Camargo Passerotti, Everson Luiz de Almeida Artifon, José Pinhata Otoch, José Arnaldo Shiomi da Cruz

**Affiliations:** 1 Universidade de São Paulo Departamento de Técnica Cirúrgica São Paulo SP Brasil Departamento de Técnica Cirúrgica, Universidade de São Paulo - USP, São Paulo, SP, Brasil;; 2 Universidade Nove de Julho Departamento de Urologia São Paulo SP Brasil Departamento de Urologia, Universidade Nove de Julho, São Paulo, SP, Brasil; 3 Serviço de Urologia Hospital Alemão Oswaldo Cruz São Paulo SP Brasil Serviço de Urologia Hospital Alemão Oswaldo Cruz, São Paulo, SP, Brasil

**Keywords:** Tadalafil, Adrenergic alpha-Antagonists, Tamsulosin, Meta-Analysis as Topic

## Abstract

**Purpose:**

Medical expulsive therapy (MET) is recommended for distal ureteral stones from 5 to 10 mm. The best drug for MET is still uncertain. In this review, we aim to compare the effectiveness of tadalafil and tamsulosin for distal ureteral stones from 5 to 10 mm in terms of stone expulsion rate (SER), stone expulsion time (SET) and the side effect profile.

**Materials and methods:**

A comprehensive literature search was conducted on MEDLINE, EMBASE, Cochrane Central Register of Controlled Trials, Scopus and Web of Science, from inception until April 2023. Only randomized controlled trials were included in the analysis.

**Results:**

Eleven publications with 1,330 patients were included. We observed that tadalafil has a higher SER (OR 0.55, CI 95% 0.38;0.80, p=0.02, I2=52%) and the same efficacy in SET (MD 1.07, CI 95% -0.25; 2.39, p=0.11, I2=84%). No differences were found when comparing side effects as headache, backache, dizziness, and orthostatic hypotension.

**Conclusion:**

Tadalafil has a higher stone expulsion rate than tamsulosin as a medical expulsive therapy for patients with distal stones from 5 to 10 mm without differences in side effects.

## INTRODUCTION

Nephrolithiasis is one of the most common diagnosed urinary diseases that mostly affects individuals between the ages of 20-40 years. The clinical presentation may include colic pain, urinary symptoms, nausea, and vomiting. Ureteral stones account for 22% of nephrolithiasis cases, with 68% being distal ureteral stones (1).

Medical Expulsive Therapy (MET) is recommended for distal stones measuring 5 to 10 mm to reduce the risk of surgical intervention and reduce the stone expulsion time (2). MET involves the use of medications that facilitate stone passage by relaxing smooth muscle, with α;-blockers, phosphodiesterase inhibitors (PDEIs), and calcium channel blockers among the commonly employed drugs (3). Nowadays, α;-blockers, such as tamsulosin, are the preferred option for MET. This recommendation, however, is based on conflicting evidence that shows limited benefits (4-6).

Network meta-analysis compared multiple alternatives for MET and, interestingly, silodosin, also an α;-blocker, seems to have the best performance as monotherapy. However, these analyses are restricted to limited number of outcomes, usually only stone expulsion rate (SER) and stone expulsion time (SET) and leave uncertain the safety profile of this interventions (7, 8).

Tadalafil, a more accessible drug, but a PDEI, has also been proposed as a viable alternative for MET. Studies and meta-analysis that compared tadalafil and tamsulosin for the treatment of distal ureteral stones from 5 to 10 mm have shown conflicting results (9, 10). The last meta-analysis about this subject was published in 2017 with 565 patients. Bai et al. (11) observed that tadalafil outperforms the tamsulosin without differences in side effects.

Given that subsequent trials (1, 12-15) have been conducted since the publication of the meta-analysis, we aim to explore the effects of tadalafil compared to tamsulosin considering new evidence. The purpose of this meta-analysis is to provide an updated assessment of the efficacy and safety of tadalafil versus tamsulosin as medical expulsive therapy for distal ureteral stones measuring 5 to 10 mm.

## MATERIALS AND METHODS

### Registration and databases search

This study was registered at Prospero CRD42023417044 (Prospero register). A search was conducted at PubMed/MEDLINE, Embase, Web of Science, Scopus, and Cochrane databases from its inception to April 2023 to identify randomized controlled trials reporting the comparison of tadalafil and tamsulosin as medical expulsive therapy for distal stones from 5 to 10mm. Our outcomes of interest were stone expulsion rate, stone expulsion time, pain episodes, analgesic use, and side effects.

### Search strategy

Tadalafil and tamsulosin and (stone or stones or nephrolithiasis or calculi or calculus) and ("randomized controlled trial" or "controlled trial" or randomized or placebo or "drug therapy" or randomly or trial or groups).

### Screening

EndNote OnlineTM was utilized to remove any duplicate studies. Two independent researchers conducted a screening of titles and abstracts to eliminate irrelevant studies. Following this process, the full text was reviewed to select the included articles. Any disagreements were solved by a third reviewer.

### Data Extraction and Quality Assessment

Data was independently extracted from the included studies by two authors. Any discrepancies among the extracted data were resolved by discussion with a third reviewer. The Rob2 score (16) was used to assess the quality of the RCTs.

## Statistical Analyses

The meta-analysis was performed by the Review Manager, version 5.4. Continuous outcomes are presented as a mean difference (MD) with 95% confidence interval (CI). Dichotomous data are presented as odds ratio (OR) with 95% CI. Pooled estimates were calculated with the random-effect model, considering that the patients came from different populations. We also performed a subgroup analysis of different doses of tadalafil. For all statistical analyses, a two-sided value of P<0.05 was considered statistically significant.

## RESULTS

**Figure 1:**
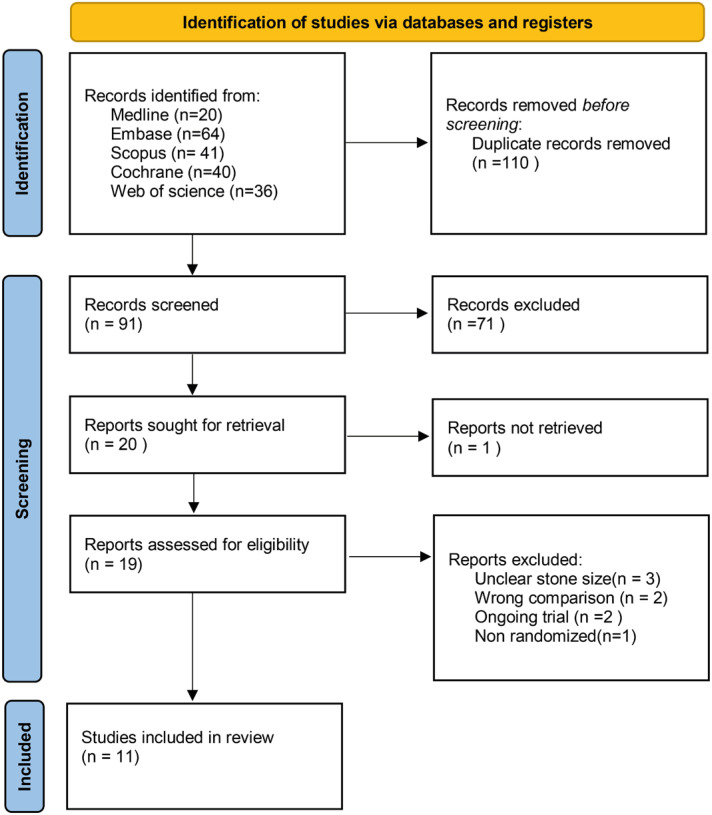
Prisma flow diagram.

The search retrieved 201 articles. After screening 11 were included in this study (Figure-1). In total, 1.330 patients were included. Most of the studies followed patients up to 4 weeks. The dosage of tamsulosin was the same across the studies(0.4mg), but 8 studies used 10mg of tadalafil and 3 studies used 5mg. Four studies lacked gender distribution (Table-1).

**Table 1:** Baseline characteristics of included studies.

Study	Tamsulosin dose (mg)	Tadalafil dose (mg)	Number of patients, Tamsulosin/Tadalafil	Mean age(y)±SD, Tamsulosin/Tadalafil	Males (%), Tamsulosin/Tadalafil	Mean stone size (mm)±SD, Tamsulosin/Tadalafil	Duration (weeks)
Abdelaal et al. (15) 2023	0.4	5	50/50	38.7/41.9^c^	70/68	6.7±1.3/6.9±1.5	4
Abishek et al., (20) 2015^b^	0.4	10	50/50	NA	NA	NA	NA
Aggarwal et al., (21) 2017^b^	0.4	10	109/109	NA	NA	NA	4
Falahatkar et al. (1) 2021	0.4	10	44/44	37.0±11.3/37.3±12	54.5/47.7	6.9±1.5/6.9±1.7	4
Goyal et al. (12) 2018	0.4	10	61/62	42.1±13.9/42.6±14.9	70.5/66.1	7.5±1.1/7.6±0.9	4
Gur et al. (13) 2021	0.4	5	48/46	41±15.9/39.0±12^a^	100/100	6.2±2.2 /6.1±1.6^a^	NA
KC et al., (22) 2016	0.4	10	41/44	31.4±12/32.1±13.3	65.9/54.5	7.1±1.2/7.1±1.5	2
Khouni et al. (14) 2022^b^	0.4	5	42/40	NA	NA	NA	6
Kumar et al., (23) 2015	0.4	10	90/90	36.4±10/37.5±13.5	68.9/74.4	7.4±1.2/7.8±1.4	4
Puvvada et al., (24) 2016	0.4	10	100/100	37.5±12.7/36.3±11.3	67/65	7.2±1.3/7.1±1.4	4
Raza et al., (25) 2016^b^	0.4	10	30/30	NA	NA	NA	4

a Estimated from median and interquartile range using the Cochrane estimator;

b Conference abstract;

c The article did not provided information about age dispersion;

RCT: randomized controlled trial;

NA not available.

### Stone expulsion

**Figure 2:**
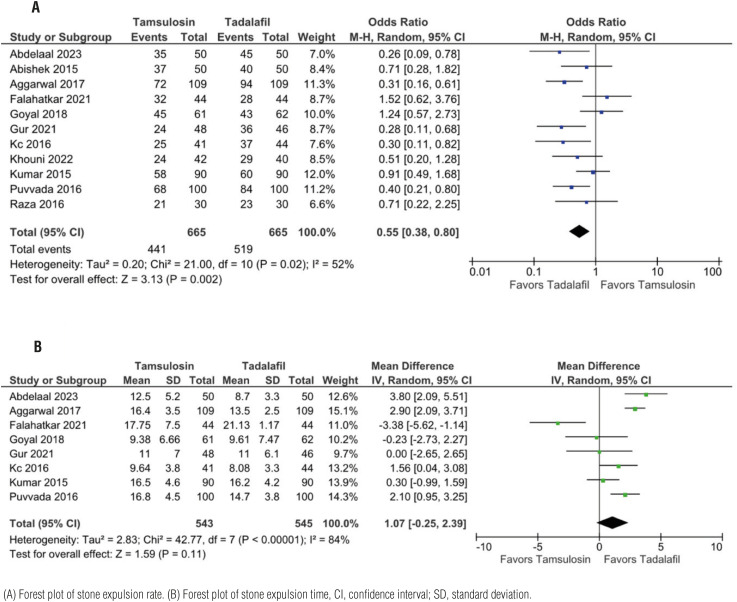
Tadalafil vs tamsulosin stone expulsion, higher SER, no difference in SET.

Overall, tadalafil was more efficient regarding our primary outcome (SER) than tamsulosin (OR 0.55, CI 95% 0.38;0.80, p=0.02, I2=52%), but no difference was observed in SET (MD 1.07, CI 95% -0.25; 2.39, p=0.11, I2=82%) (Figure-2).

### Pain episodes and analgesic use

**Figure 3:**
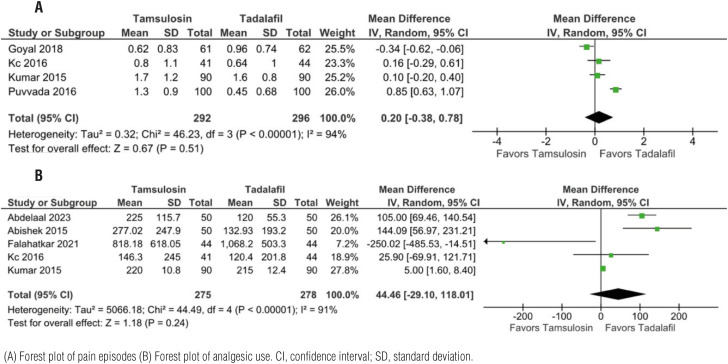
Tadalafil vs tamsulosin no difference in pain episodes or analgesic use.

Only 4 RCT assessed pain episodes, with 292 patients in the tamsulosin group, and 296 patients in the tadalafil group. No statistical significance was observed when comparing pain episodes (OR 0.20 CI 95% -0.38; 0.78, p=0.51, I=94%) or analgesic use (MD 44.46, CI 95% -29.10; 118.01, p=0.24, I=91%) (Figure-3). Regarding analgesic use, all authors used oral diclofenac as analgesic, only KC et al. used aceclofenac.

### Side effects

**Figure 4:**
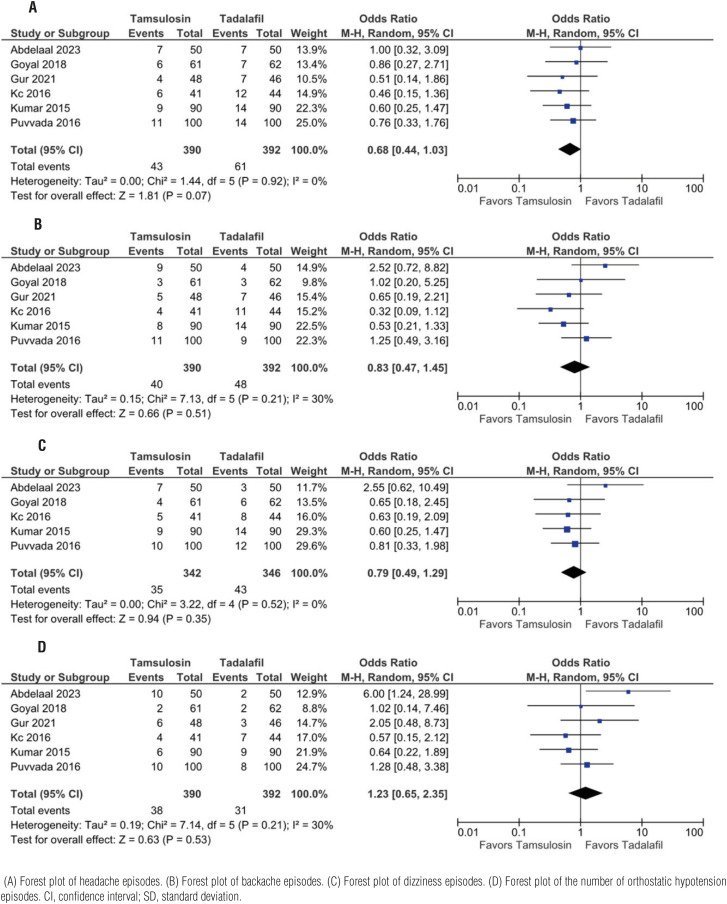
Tadalafil vs tamsulosin no difference in side effects.

No difference was observed among all the assessed side effects. Six studies reported headache(OR 0.68, CI 95% 0.44;1.03, p=0.92, I2=0%), backache(OR 0.83, CI95% 0.47; 1.45, p=0.51, I2=30%), and orthostatic hypotension(OR 1.23, CI95% 0.65;2.35, p=0.53, I2=30%) as side effects while only five reported dizziness (OR 0.79, CI95% 0.49;1.29, p=0.35, I2=0%) (Figure-4).

### Subgroup analyses

We performed a subgroup analysis of SER and SET among different doses of tadalafil (5mg and 10 mg) (Appendix-1). We found that the subgroup receiving a 5mg showed a higher benefit in terms of SER (OR 0.34, CI 95% 0.19;0.59, p=0.0001, I2=0%) compared to the subgroup receiving a 10mg dose (OR 0.65, CI 95% 0.42;1.00, p=0.05, I2= 56%). No differences were observed when comparing SET within both subgroups, 5mg (MD 2.04, CI95% -1.67;5.75, p=0.28, I2=82%) and 10mg (MD 0.76, CI95% -0.76;2.27, p=0.33, I2=86%).

## DISCUSSION

Our meta-analysis included 1330 patients from 11 RCTs comparing tamsulosin and tadalafil in cases with distal ureteral stones from 5 to 10mm. We observed that tadalafil has the same SET and even a greater SER than tamsulosin, without differences in side effects (headache, dizziness, backache, and orthostatic hypotension).

Medical expulsive therapy (MET), typically using α;-blockers, is an off-label recommendation, supported by urological societies, to facilitate the passage of distal ureteral stones sized 5-10 mm (2, 3). This recommendation is based on a pooled analysis on 27 studies that shows a SER of 77.3% of α;-blockers, compared to 54.4% of placebo (2). However, this recommendation is contentious due to contradictory evidence from randomized controlled trials, demonstrating limited benefits of this therapy (4-6). In this regard, tadalafil, has also been suggested as a potential alternative, with concerns about adverse events and uncertainties about its efficacy (17).

The largest previous meta-analysis, conducted by Bai et. al. (11), included 565 patients from 4 RCTs. Bai et. al. described that tadalafil outperforms tamsulosin not only when comparing SER (without differences in side effects) but also when comparing analgesic use and SET. Indeed, the last two comparisons were made applying the fixed effect model. This model is a controversial approach in statistics that increases the chance of false positive results (18). In our study all the comparisons were done using the random effects model.

Curiously lower doses of tadalafil seem to be more effective. Even if a statistical artifact seems to be the most reasonable explanation to this finding, as it goes against the dose-response principle, it is also biologically plausible that excessive smooth muscle relaxation can hinder the stone expulsion process, as observed in some studies comparing antispasmodics with placebo (8).

A more modern approach to establish the best MET is the use of network meta-analysis (NMA) (7, 8). The most recent was conducted by Sharma et. al. in 2021 (7). This NMA compared 50 RCTs involving various interventions such as α;-blockers, PDEi, and CCB (calcium channel blockers). The outcomes were exclusively SER and SET.

Even if it claimed that the two most effective options for MET are a combination of naftodipil and steroids (resulting in the highest SER) or a combination of tadalafil and silodosin (resulting in the shortest SET) the absence of side effect assessment limits its clinical significance (7). It also included patients with stones smaller than 5mm, which represents a subgroup where the benefits of MET are not clearly established (2, 3). Additionally, the exclusion of conference abstracts from the analysis raises concerns about potential publication bias.

Based on our clinical experience dealing with patients with urological conditions in an ambulatory setting, in Brazil, we observe that MET with tadalafil has a lower cost than MET with tamsulosin and we believe that it could be true for other centers, which strengthens our recommendation. However, our study lacks a cost-effective analysis as this information was not provided by the included studies.

**Figure 5:**
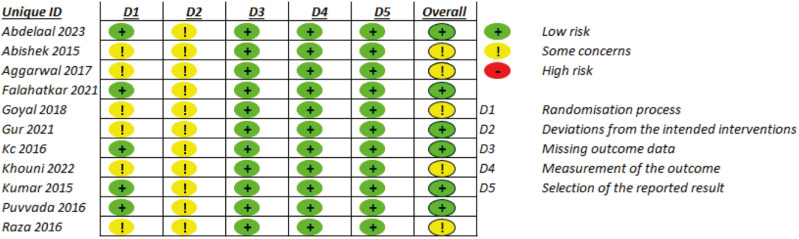
Risk of bias of the included studies.

Our study has limitations. Included articles lacked information regarding time from beginning of the symptoms to introduction of MET, and regarding use of additional medications that could affect the efficacy of the treatment. As a confounding factor, the impact of tadalafil on frequency of sexual intercourse, which also have a potential role in the stone expulsion, was not assessed either (19). Although articles presented low bias, the absence of description of the allocation concealment in all included trials may result in some undetected bias (Figure-5).

Finally, our study leaves unanswered questions as the safety and efficacy of combined therapy with tamsulosin and tadalafil for distal ureteral stones and the ideal dosage of tadalafil for MET.

## CONCLUSIONS

Tadalafil has a higher stone expulsion rate than tamsulosin as a medical expulsive therapy for patients with distal stones from 5 to 10 mm without differences in side effects.
